# Use of family practice journals in Journal Clubs: a study from the Middle East

**DOI:** 10.3399/bjgpopen17X100953

**Published:** 2017-05-17

**Authors:** Basem Saab, Rim Taleb, Adel Al Bari, Hashim Al-Sayed, Issam Shaarani

**Affiliations:** 1 Professor of Family Medicine & Programme Director, Department of Family Medicine, American University of Beirut Medical Center, Beirut, Lebanon; 2 Chief Resident & Family Physician, Department of Family Medicine, American University of Beirut Medical Center, Beirut, Lebanon; 3 Assistant Professor of Family Medicine, Family Practice Residency Program, Ministry of Health, Manama, Kingdom of Bahrain; 4 Associate Professor of Family Medicine, Department of Medicine, Primary Health Care Corporation, Doha, Qatar; 5 Senior Lecturer of Family Medicine, Department of Clinical Sciences, Beirut Arab University, Beirut, Lebanon

**Keywords:** family practice, journal, journal club, Middle East

## Background and method

A journal club is a common educational activity that is used to teach residents skills in practicing evidence-based medicine.^1^ It is said that Sir William Osler introduced a journal club in 1875 to McGill University,^2,3^ but others think it was started earlier in England and Germany.^4^ Several bodies highlight the importance of appraising research critically for medical training.^2,4^

We have noticed that most of the time family medicine residents in our programmes use journals from other specialties in our journal club and, to the best of our knowledge, there is no information available on the journals that they generally use for this activity. We conducted a survey to explore whether residents in four programmes in the Middle East (Bahrain, Lebanon, Oman, and Qatar) are aware of and consider major family practice journals while preparing for their journal club presentations.

All family medicine residents who present in the journal club in these programmes were asked to complete a LimeSurvey (an online survey tool). Journals included those that specialise in family practice, publish original research in English, and had an impact factor of ≥1.5 in 2014. Three reminders were sent by e-mail to improve response rate.

## Results

The journals that satisfied the inclusion criteria were the *Journal of the American Board of Family Medicine* (*JABFM*), the *British Journal of General Practice* (*BJGP*), *Annals of Family Medicine* (*Ann Fam Med*), *Family Practice* (*Fam Pract*), and *BMC Family Practice* (*BMC Fam Pract*). The *Scandinavian Journal of Primary Care* satisfied the inclusion criteria, but it was dropped from the list as only one resident was aware of it.

Sixty-one of 147 residents completed the survey (response rate of 42%). Most of the participating residents (92%) were aged 25–30 years. Eighty-two per cent of the residents were female and 62% had free online access in their training institution or hospital.

Eighty per cent of the participating residents stated that they select articles pertaining to subjects of interest and 69% choose articles that have a bearing on the management of patients seen earlier. Only a small minority of the residents (7%) reported to present articles suggested by drug representatives.

The top three journals the residents were aware of included *JABFM* (89%), the *BJGP* (53%), and *Fam Pract* (41%) and 46–82% of residents who were aware of the leading family practice journals would consider presenting an article from one of these journals in their journal club. With the exception of the *JABFM*, the residents seldom (every 3–6 months) read the included journals.

The *JABFM* was reported to be the most interesting journal (95%) followed by *BJGP* (89%). Eighty-three per cent of residents who were aware of the *Ann Fam Med* and *Fam Pract* found these journals interesting.

## Discussion

Awareness of family practice journals that publish original research and have an impact factor ≥1.5 was low among the residents. Only 34% and 53% were aware of *Ann Fam Med* and the *BJGP* respectively. Both journals have the highest impact factors in family medicine, and the *BJGP* has been in circulation for more than six decades. Programme directors and family medicine faculties in the surveyed departments need to make an effort to improve the noted deficiency; 41% of the residents indicated that suggestions by faculty members have a bearing on the journals they select for presentation at their journal club. The respective Editorial Boards need to promote their journals; one possible way is to disseminate what they publish directly by email to residents as 39% reported that they use articles received electronically in their journal club. There is also a need to understand better why residents in the four family medicine programmes have relatively low interest in selecting articles from journals highly pertinent to their discipline.

The *JABFM* was the most read journal; 89% of the responders reported to know the journal and 82% would consider it for their journal club activity presentations. One possible explanation for this popularity is that three of the involved programmes (Bahrain, Oman, and Lebanon) currently assess their residents using the in-training exam that is prepared by the American Board of Family Medicine or have used this previously for assessment.

## References

[1] Mohr NM, Stoltze AJ, Harland KK (2015). An evidence-based medicine curriculum implemented in journal club improves resident performance on the Fresno test. J Emerg Med.

[2] Lee AG, Boldt HC, Golnik KC (2005). Using the journal club to teach and assess competence in practice-based learning and improvement: a literature review and recommendation for implementation. Surv Ophthalmol.

[3] Linzer M (1987). The journal club and medical education: over one hundred years of unrecorded history. Postgrad Med J.

[4] Royal College of General Practitioners (2017). The RCGP Curriculum: Professional & Clinical Modules. 2.01–3.21 Curriculum Modules. http://www.rcgp.org.uk/training-exams/gp-curriculum-overview/~/media/Files/GP-training-and-exams/Curriculum-2012/RCGP-Curriculum-modules.ashx.

